# Bottom-up construction of low-dimensional perovskite thick films for high-performance X-ray detection and imaging

**DOI:** 10.1038/s41377-024-01521-2

**Published:** 2024-07-23

**Authors:** Siyin Dong, Zhenghui Fan, Wei Wei, Shujie Tie, Ruihan Yuan, Bin Zhou, Ning Yang, Xiaojia Zheng, Liang Shen

**Affiliations:** 1 Sichuan Research Center of New Materials, Institute of Chemical Materials, China Academy of Engineering Physics, Shuangliu, Chengdu China; 2 State Key Laboratory of Integrated Optoelectronics, College of Electronic Science and Engineering, International Center of Future Science, Jilin University, Changchun, China

**Keywords:** X-rays, Imaging and sensing

## Abstract

Quasi-two-dimensional (Q-2D) perovskite exhibits exceptional photoelectric properties and demonstrates reduced ion migration compared to 3D perovskite, making it a promising material for the fabrication of highly sensitive and stable X-ray detectors. However, achieving high-quality perovskite films with sufficient thickness for efficient X-ray absorption remains challenging. Herein, we present a novel approach to regulate the growth of Q-2D perovskite crystals in a mixed atmosphere comprising methylamine (CH_3_NH_2_, MA) and ammonia (NH_3_), resulting in the successful fabrication of high-quality films with a thickness of hundreds of micrometers. Subsequently, we build a heterojunction X-ray detector by incorporating the perovskite layer with titanium dioxide (TiO_2_). The precise regulation of perovskite crystal growth and the meticulous design of the device structure synergistically enhance the resistivity and carrier transport properties of the X-ray detector, resulting in an ultrahigh sensitivity (29721.4 μC Gy_air_^−1^ cm^−2^) for low-dimensional perovskite X-ray detectors and a low detection limit of 20.9 nGy_air_ s^−1^. We have further demonstrated a flat panel X-ray imager (FPXI) showing a high spatial resolution of 3.6 lp mm^−1^ and outstanding X-ray imaging capability under low X-ray doses. This work presents an effective methodology for achieving high-performance Q-2D perovskite FPXIs that holds great promise for various applications in imaging technology.

## Abstract (web summary)

The quasi-two-dimensional perovskite films were successfully fabricated through a precisely controlled crystallization process by utilizing a mixed atmosphere of CH_3_NH_2_ and NH_3_, resulting in the development of an ultra-sensitive perovskite/TiO_2_ heterojunction X-ray detector.

## Graphical abstract

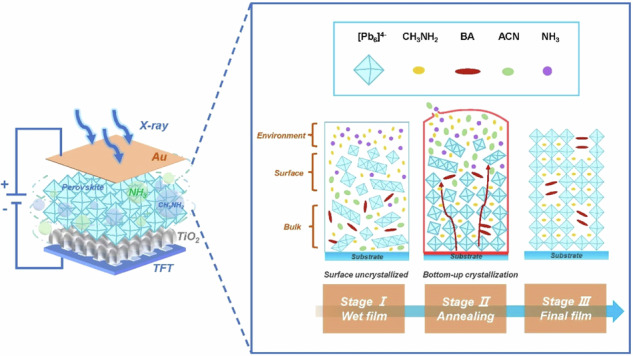

## Introduction

X-ray detection is widely utilized in medical imaging, product inspection applications, and scientific research fields^1–3^. Metal halide perovskites have emerged as highly promising materials for exceptionally sensitive X-ray detectors owing to their remarkable detection properties, superior X-ray stopping power, large mobility-lifetime (*μτ*) products, tunable bandgap characteristics, and reduced free charge carrier density^3–8^. X-ray detectors utilizing 3D perovskite materials have demonstrated exceptional sensitivity (>10^5^ μC Gy_air_^−1^ cm^−2^) and an impressively low detection limit (~1 nGy_air_ s^−1^)^9,10^. However, the well-known phenomenon of ion migration poses a significant challenge to achieving long-term stability in these materials, thereby impeding their widespread commercialization^11^. It has been observed that by incorporating spacer layers and harnessing quantum well effects through the introduction of long-chain insulated cations into low-dimensional perovskite structures, it is possible to effectively suppress ion migration and enhance device stability^12–17^. In particular, the Q-2D perovskite exhibits remarkable photoelectric properties and stability by carefully selecting spacer cations and precisely controlling the number of [BX_6_]^−^ layers, thus making it highly promising for future commercial applications^16^.

The strong penetrability of X-rays makes their weak interaction with detection materials. Thus, perovskites with a thickness of hundreds of micrometers are needed to ensure an effective utilization of incident X-rays. Solution preparation is a convenient method for preparing perovskite due to its simplicity and short preparation period^18^. Currently, the primary methods for preparing perovskite films are spin-coating and blade-coating^19,20^. However, these traditional approaches face challenges in achieving such thicknesses. Firstly, due to the limited solid content and wet film thickness, films obtained through these methods typically have thicknesses below 10 micrometers, resulting in poor X-ray absorption capabilities that do not meet the requirements of X-ray detection applications. Secondly, the crystallization of the perovskite precursor solution on the surface can impede solvent escape at the buried interface, leading to holes and cracks in annealed thick films^21,22^. Thus, a novel synthesis route for growing large-scale halide perovskite thick films directly on a thin-film transistor (TFT) substrate is badly needed for high-performance perovskite X-ray detectors^23^.

In this study, we developed a novel approach to control the crystallization of Q-2D perovskite in a mixed atmosphere consisting of NH_3_ and CH_3_CH_2_. We employed a high solid content liquid perovskite, BA_2_MA_9_Pb_10_I_31_·xCH_3_NH_2_, prepared through solid-liquid conversion by CH_3_NH_2_ intercalation, which offers advantages of high solid content, fluidity, easy processing, and easy solidification through the rapid release of CH_3_NH_2_. Through precise modulation of the crystallization driving force in the wet perovskite film using a mixed NH_3_ and CH_3_CH_2_ atmosphere, we achieved bottom-up crystallization of perovskite, leading to successful preparation of high-quality perovskite films exceeding 100 μm in thickness. Additionally, by constructing a TiO_2_-perovskite heterojunction, we effectively reduced the dark state current density and enhanced carrier extraction efficiency in X-ray detectors. The results demonstrate that our Q-2D perovskite heterojunction X-ray detector exhibits an ultrahigh sensitivity (29721.4 μC Gy_air_^−1^ cm^−2^), surpassing previously reported low-dimensional perovskite polycrystalline X-ray detectors, and achieves a low detection limit of 20.9 nGy_air_ s^−1^. More importantly, the flat panel X-ray imager (FPXI) achieves clear X-ray images at low X-ray doses and shows a spatial resolution of 3.6 lp mm^−1^ (0.72 lp pix^−1^) at modulation transfer function (MTF) = 0.2, which is among the highest spatial resolution for all the reported perovskite FPXIs.

## Results

**Fig. 1:**
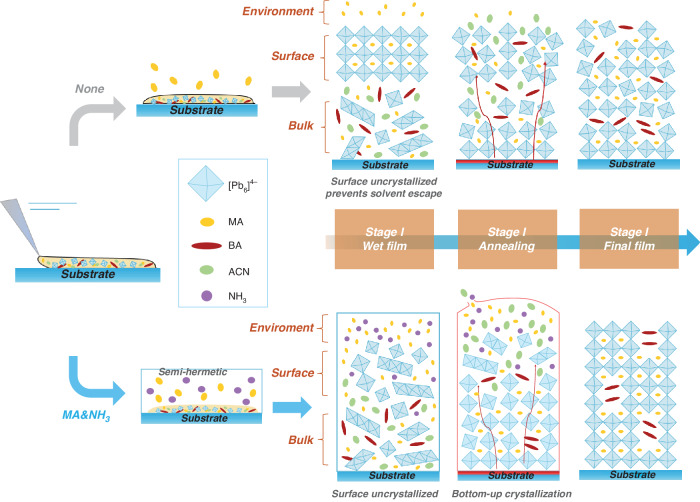
Schematic diagram illustrating the mechanism of film formation. Comparison of the normal perovskite fabrication process and the atmosphere-assisted process, highlighting the distinctive ability of the latter to fabricate high-quality thick, dense perovskite films

The preparation of a thick perovskite film relies on a high concentration of the precursor solution. The liquid perovskite of BA_2_MA_9_Pb_10_I_31_ was prepared through a solid-liquid conversion method utilizing CH_3_NH_2_ gas^24^. Additionally, acetonitrile (ACN) was introduced in an appropriate quantity to fulfill the requirements for blade-coating. Figure. 1 illustrates the rationale for developing the atmosphere assist method by comparing with direct annealing method to fabricate perovskite films. We first scrape liquid perovskite onto the substrate to obtain a fresh film, the wet film preferentially solidifies near the surface through homogeneous nucleation and growth as solvent evaporation, which preventing the escape of remaining CH_3_NH_2_ and ACN. During the annealing process, the film quality deteriorates as a result of the solvent’s rapid evaporation and the crystals’ accelerated growth. However, when the wet film is exposed to CH_3_NH_2_ gas, it does not reach saturation at the top surface and crystallization is inhibited. Therefore, crystallization occurs from the bottom to up during annealing process, and the solvent can escape effectively. Notably, we employed a semi-enclosed environment to ensure gradual discharge of excess solvent and gas during the heat annealing process, thereby facilitating complete crystallization of the perovskite surface.

**Fig. 2:**
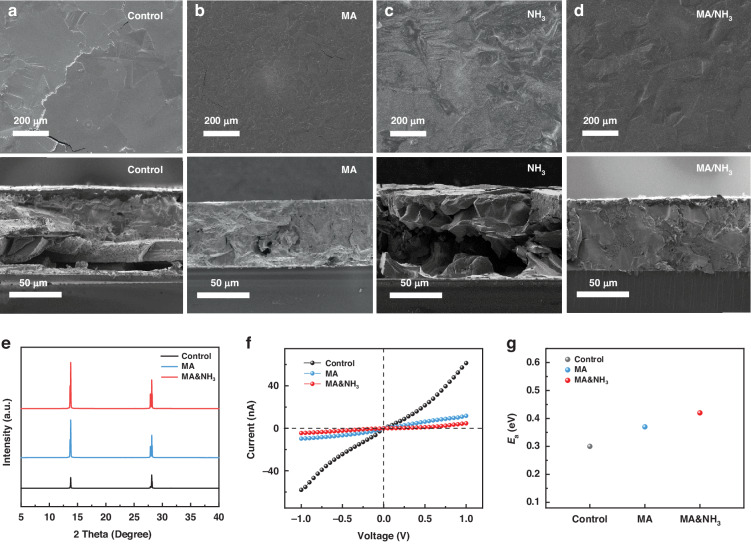
Microstructure and electronic characteristics of the perovskites. SEM images for BA_2_MA_9_Pb_10_I_31_ films prepared under different conductions: (**a**) Control, (**b**) CH_3_NH_2_, (**c**) NH_3_, and (**d**) CH_3_NH_2_/NH_3_ atmospheres. **e** X-ray diffraction patterns of BA_2_MA_9_Pb_10_I_31_ films; (**f**) Resistivity of BA_2_MA_9_Pb_10_I_31_ films; (**g**) Activation energy for ion migration of the BA_2_MA_9_Pb_10_I_31_ films prepared under various conductions

The scanning electron microscope (SEM) images in Fig. 2 present the surface and cross-section morphology of the Q-2D perovskite films. It is observed that annealing the perovskite film in a CH_3_NH_2_ atmosphere results in a more uniform and dense film compared to those obtained by direct annealing (Fig. 2a, b). This implies that the presence of CH_3_NH_2_ gas hinders the initial crystallization of the film surface during annealing, while the bottom-up crystallization process effectively mitigates any adverse effects on film quality caused by solvent volatilization. We found that when NH_3_ gas was used, the grain size of the perovskite films was obviously increased, which indicates that NH_3_ can effectively delay the crystallization rate of perovskite. However, the perovskite film still tends to crystallize on its surface first, resulting in numerous voids due to the solvent evaporation process during annealing (Fig. 2c). To realize high-quality dense perovskite film with large grains, a mixed CH_3_NH_2_ and NH_3_ atmosphere was used. As shown in Fig. 2d, the perovskite film obtained under a certain proportion of CH_3_NH_2_ and NH_3_ mixed atmosphere is relatively dense, and the cracks and holes on its surface and cross section are less, indicating that the coordination of mixed atmosphere can regulate the crystal growth process of perovskite and make it crystallize slowly from bottom to up, and obtain high-quality films. More importantly, the method enables the fabrication of perovskite films with controlled thickness ranging from tens to hundreds of micrometers by modulation of the precursor solution volume and atmosphere (Fig. S1). We further fabricated perovskite films on 5 × 5 cm^2^ quartz glass substrates. The results presented in Fig. S2 demonstrate a clear enhancement in the quality of perovskite films achieved through controlled crystallization in a mixed atmosphere. Simultaneously, the steady-state photoluminescence (PL) of the perovskite film prepared with a mixed atmosphere further supports its superior quality (Fig. S3).

In order to investigate the influence of atmosphere on the crystallization behavior of Q-2D perovskite, we further characterized the phase structure of Q-2D perovskite film under different atmospheres, and the XRD results are shown in Fig. 2e. The perovskite films prepared under MA and mixed atmosphere both exhibited the higher crystallinity, thereby demonstrating that atmosphere effectively retards the crystal growth process and facilitates the formation of films with larger grain sizes, which was consistent with the results of SEM. Notably, the perovskite films synthesized in an NH_3_-rich atmosphere exhibited diffraction peak corresponding to NH_4_PbI_3_, indicating that NH_3_ can incorporate into the lattice and form the NH_4_PbI_3_ phase (Fig. S4). However, no diffraction peak corresponding to the NH_4_PbI_3_ was observed in the perovskite film prepared under a mixed atmosphere, indicating the complete removal of NH_3_ following annealing. Therefore, mixing a certain proportion of NH_3_ can effectively delay the crystal growth rate to improve the quality of perovskite film by forming the NH_4_PbI_3_ intermediate phase.

As shown in Fig. S5, the absorption edge of all BA_2_MA_9_Pb_10_I_31_ films is ~790 nm, and the corresponding band gap is ~1.59 eV, which is in the ideal band gap range of X-ray detectors at room temperature^25^. The *E*_g_ of the BA_2_MA_9_Pb_10_I_31_ film was determined by the Tauc equation for the direct band gap:

$${(\alpha {hv})}^{2}={\rm{A}}({hv}-{E}_{{\rm{g}}})$$

where *α* is the absorption index, *h* is the Planck constant, *v* is the frequency, and A is a constant. All BA_2_MA_9_Pb_10_I_31_ films have similar band gaps, which is same as the XRD results, indicating that no NH_4_PbI_3_ remains after annealing.

A high resistivity is essential to reduce the noise of the X-ray detector. We further characterized the resistivity of BA_2_MA_9_Pb_10_I_31_ film. As shown in Fig. 2f, the bulk resistivity of the Q-2D perovskite obtained by Control, CH_3_NH_2_ atmosphere, and CH_3_NH_2_/NH_3_ atmosphere are 1.80 × 10^7^ Ω cm, 8.65 × 10^7^ Ω cm and 2.48 × 10^8^ Ω cm, respectively. The film prepared in mixed atmosphere has a highest resistivity, which is due to fewer cracks and defects inhibit leakage current.

The ideal X-ray detection material should have a high activation energy (*E*_a_) for ion conduction, thereby effectively restricting ion migration and enhancing the stability of the device^14^. We calculated the *E*_a_ of BA_2_MA_9_Pb_10_I_31_ and MAPbI_3_ films (prepared in mixed atmosphere) by temperature-dependent conductivity curves (Fig. S6). The *E*_a_ value of Q-2D perovskite film prepared in Control, CH_3_NH_2_ atmosphere, and CH_3_NH_2_/NH_3_ atmosphere is 0.365 eV, 0.589 eV, and 0.632 eV, respectively (Fig. 2g), and higher than that of 0.301 eV of MAPbI_3_. The higher *E*_a_ of Q-2D perovskites means more difficult ion migration in BA_2_MA_9_Pb_10_I_31_ films, and it allows the utilization of larger bias to enhance carrier drift length and improve carrier collection. The results also demonstrate that the mixed atmosphere can effectively enhance the quality of Q-2D perovskite films, further inhibit ion migration, and improve the stability of the device.

Subsequently, we investigate the *μτ* product in BA_2_MA_9_Pb_10_I_31_ films by using photoconductivity method. Figure S7 shows the typical bias-dependent photoconductivity of BA_2_MA_9_Pb_10_I_31_ film under negative and positive bias. The *μτ* product of the Q-2D perovskite obtained by fitting the modified Hecht equation^26^:

$${{J}}_{{\bf{s}}}{=}\frac{{{J}}_{{\bf{0}}}{\mu}{\tau}{V}}{{{d}}^{{2}}}\left[{1}{-}{\exp }{\left({-}\frac{{{d}}^{{2}}}{{\mu }{\tau }{V}}\right)}\right]$$

where *J*_s_ is the signal current density, *J*_0_ is the saturated photocurrent density, *d* is the thickness of the film, *V* is the applied bias voltage, *μ* is the carrier mobility and *τ* is the average carrier lifetime. The Q-2D perovskite prepared in the mixed atmosphere have higher *μτ* product than that of CH_3_NH_2_ atmosphere, indicating the higher quality of film can improves the charge transport performance of device. The *μ*_e_*τ*_e_ of the mixed atmosphere perovskite film is 7.81 × 10^−5^ cm^2^ V^−1^, which is significantly higher than that of 10^−7^ cm^2^ V^−1^ of commercial a-Se^27,28^.

To further evaluate the charge transfer performance of Q-2D perovskite, we characterized the mobility of charge carriers in these Q-2D perovskites using the time of flight (TOF) method. The transient photocurrent response of the BA_2_MA_9_Pb_10_I_31_ device under different external electric field, from which the transit time (*t*_tr_) of the carriers can be obtained by the intersection of the asymptote from the platform and the tail line in the double logarithmic diagram. The mobility is then obtained by the following formula:

$$\mu =\frac{{d}^{2}}{V{t}_{{tr}}}$$

where *V* is the bias, and *d* is the thickness of the film. The mobility for holes and electrons can be obtained by changing the polarities of the applied bias. The carrier mobility of perovskite prepared in mixed atmosphere is 19.3 cm^2^ V^−1^ s^−1^, surpassing the value of 18.1 cm^2^ V^−1^ s^−1^ obtained for CH_3_NH_2_ under the same bias (Fig. S8), which further proves that mixed atmosphere can effectively enhance the carrier transport performance of the Q-2D perovskite.

High attenuation efficiency of the perovskite is crucial for excellent detection performance of the X-ray detector which can reduce both the needed thickness of the absorber and the challenges in collecting low-dose X-ray-generated carriers. Figure S9a shows the absorption spectra of BA_2_MA_9_Pb10I_31_, CZT, Si, MAPbBr_3_, and MAPbI_3_ in the photon energy range of 0.01–10 MeV. Due to the large average atomic number and high density, BA_2_MA_9_Pb_10_I_31_ has a good radiation attenuation ability and is favorable for complete X-ray absorption. Figure S9b shows that the attenuation efficiency of different materials for 40 keV X-ray photons changes with thickness. The attenuation efficiency of BA_2_MA_9_Pb_10_I_31_ and CZT and 3D perovskite is similar and much higher than that of Si, which indicates BA_2_MA_9_Pb_10_I_31_ can achieve similar or better X-ray photon absorption than commercial materials with same thickness.

**Fig. 3:**
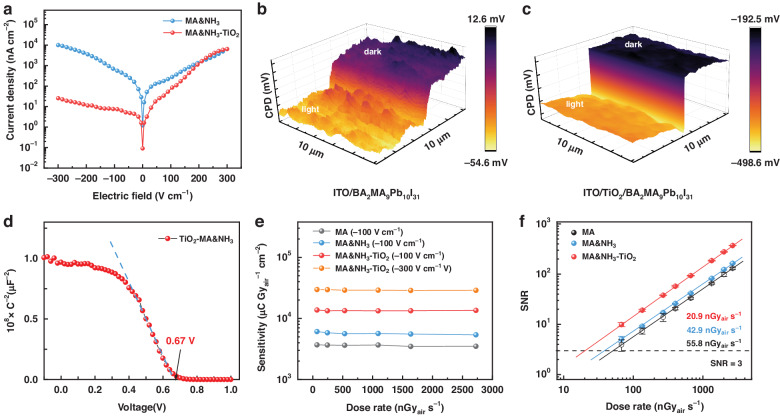
Effect of TiO_2_ functional layer on the device performance. **a** Dark *IV* curves for devices with/without TiO_2_; (**b**) CPD maps (KPFM images) of ITO-based; and (**c**) TiO_2_-based devices under dark and light conditions. The Surface CPD of light conditions were recorded under 20 mW cm^−2^ white light illumination. **d** Mott-Schottky curve calculates the *V*_bi_ of TiO_2_-Q-2D perovskite X-ray detector; (**e**) The sensitivity of the X-ray detectors with dose rate from 70 nGy_air_ s^−1^ to 2726 nGy_air_ s^−1^. **f** X-ray dose rate-dependent SNR of the X-ray detectors under an electric field of −100 V cm^−1^, and the error bars represent the standard deviation

Currently, the prevailing configuration for X-ray detectors involves an electrode/perovskite/electrode structure, and there are less reports on heterojunction X-ray detectors constructed by charge transport layer and perovskite. Heterojunction is predicted as an effectively strategy to improve the charge transport features in perovskite X-ray detectors, thereby improving sensitivity of the device^29–33^. We next integrate TiO_2_ with ITO substrates at low temperatures to boost the performance of the device (Fig. S10). The *IV* curve of TiO_2_-Q-2D perovskite films exhibited a pronounced rectification phenomenon (Fig. 3a). Under negative bias, the dark current density was hundreds of times lower than that observed without TiO_2_. The TiO_2_-Q-2D perovskite heterojunction can effectively reduce device noise by increasing the resistivity under reverse bias, leading to better detection limits.

We further studied the effect of TiO_2_ layer on charge extraction properties by Kelvin probe force microscopy (KPFM). Figure 3b, c shows the surface contact potential difference (CPD) of Q-2D perovskite in dark and light states with different substrates. Compared with the perovskite integrated on the ITO, the dark state CPD of perovskite with TiO_2_ layer decreases from close to 10 mV to about −190 mV, which indicates exist an inherent electric field in the perovskite/TiO_2_ heterojunction and promotes the separation of charge, making the perovskite surface have more P-type semiconductor properties. In addition, the CPD of TiO_2_-Q-2D perovskite changes more obvious in light and dark conditions, represent the faster separated of photogenerated carrier. The rectification of the *IV* curve also indicates the presence of a built-in electric field (*V*_bi_) in TiO_2_-Q-2D perovskite X-ray detector. Therefore, the capacitance-voltage(*C*-*V*) curve was measured to investigate the *V*_bi_ of the TiO_2_-Q-2D perovskite film according to the Mott-Schottky relationship (Fig. 3d). The *V*_bi_ value of TiO_2_-Q-2D perovskite device is 0.67 V, leading to more efficient charge separation. Moreover, time-resolved PL (TRPL) spectra were measured to gain insight into the electron transport performance of devices TiO_2_-Q-2D perovskite (Fig. S11). The average lifetime (*τ*_ave_) of the Control (Q-2D perovskite/quartz glass) and TiO_2_-Q-2D perovskite film is 113.4 ns and 55.8 ns, respectively, which represent the faster electron transfer in TiO_2_-Q-2D perovskite devices. Therefore, the improved carrier separation and transmission performance from the device structure design leads to higher sensitivity of the TiO_2_-Q-2D perovskite X-ray detector.

The sensitivity and the limit of detection (LoD) are the two most crucial performance metric for X-ray detectors. The sensitivity describes the ability of a detector to convert X-ray photons to electronic signals. And LoD is the lowest dose rate at which the detector still produces a signal-to-noise ratio (SNR) of 3, indicating the lowest dose rate required for high-resolution images acquisition^34^. We first prepared a film detector with Au/BA_2_MA_9_Pb_10_I_31_/ITO vertical device structure, and characterized the X-ray detection performance at the X-ray dose rate range of 70–2726 nGy_air_ s^−1^, and the average X-ray photon energy is 42 keV. At −100 V cm^−1^ electric field, the X-ray sensitivity and detection limit of Q-2D perovskite X-ray detectors prepared in CH_3_NH_2_/NH_3_ mixed atmosphere and CH_3_NH_2_ atmosphere are ~ 6000 μC Gy_air_^−1^ cm^−2^, ~ 3700 μC Gy_air_^−1^ cm^−2^, and 44.2 nGy_air_ s^−1^, 55.8 nGy_air_ s^−1^ (Fig. 3e, f), respectively, which proves that the mixed atmosphere regulating perovskite crystallization can effectively improve the performance of X-ray detectors.

Due to the enhanced electron transport performance and resistivity of the heterojunction structure, the sensitivity and LoD of TiO_2_-Q-2D perovskite X-ray detector are approximately 13,700 μC Gy_air_^−1^ cm^−2^ and 20.9 nGy_air_ s^−1^ under −100 V cm^−1^ (Fig. 3e, f), which is much better than that device without TiO_2_. Additionally, the incorporation of a TiO_2_ layer enhances the anti-bias performance of the X-ray detector, enabling it to operate at a higher electric field (-300 V cm^−1^) and achieving a recording sensitivity of 29721.4 μC Gy_air_^−1^ cm^−2^ for the Q-2D polycrystalline perovskite detector. The signal current shows linearly increase with X-ray dose-rate (Fig. S12c). Figure S12d shows the bias-dependent sensitivity of the TiO_2_-Q-2D perovskite device under varying X-ray dose rates. We further calculate and compare the detection efficiency of different devices. Theoretical sensitivity *S*_0_ by^26^:

$${S}_{0}=\frac{(\varphi /X)\bar{E}\beta }{{W}_{\pm }}e\eta$$

where *φ*/X is the number of photons per unit of exposure, $\bar{E}$ is the mean energy of the X-ray photons, *β* is the energy absorption efficiency of X-rays, *W*± is the mean ionization energy to create an electron−hole pair, *e* is the elemental electron charge, and *η* is the charge collection efficiency of the

device. The *W*± for BA_2_MA_9_Pb_10_I_31_ is calculated to be 4.61 eV with an *E*_g_ of 1.59 eV according to the empirical model:

$${W}_{\pm }=2{E}_{g}+1.43{eV}$$

The mean energy *E̅* of the X-ray photons is 42 keV in this work. *φ*/X is ~545,106 photons mm^−2^ mR^−1^, which equals 6.22 × 10^12^ photons cm^−2^ Gy_air_^−1^ (1 mR = 8.76 × 10^−6^ Gy_air_). *β* and *η* are assumed to be 100% to obtain the theoretical detection sensitivity. The *S*_0_ is 9070.8 μC cm^−2^ Gy_air_^−1^ for the BA_2_MA_9_Pb_10_I_31_ device. Then the detection efficiency of 151.5% is calculated by *S*/*S*_0_ when the TiO_2_-detector under -100 V cm^−1^, higher than that of the CH_3_NH_2_/NH_3_ (67.2%) and CH_3_NH_2_ (40.7%) devices without TiO_2_, which indicated the atmosphere-assisted control of perovskite crystallization and device structure design significantly improved the detection performance of BA_2_MA_9_Pb_10_I_31_ X-ray detector.

Notably, the built-in electric field enables the detector to have a significant X-ray response without external bias, and achieves a sensitivity of ~1500 μC Gy_air_^−1^ cm^−2^ and a LoD of ~40.1 nGy_air_ s^−1^ at 0 V cm^−1^ (Fig. S13). Through the design of heterojunction devices, the realization of self-driven X-ray imaging is also an important direction of future development^35,36^.

In addition to the X-ray detection performance, the operational stability of the detector is the other crucial aspect governing its application. Therefore, we first evaluate the dark current stability of X-ray detectors under specific operating electric fields. As shown in Fig. S14, the dark current of device does not shift significantly within 1 h under −100 V cm^−1^ electric field, which is attributed to weak ion migration in Q-2D perovskite. Figure S14b further presents the operation stability of the device under an operating bias of -100 V cm^-1^ and high X-ray dose rate of 72.5 mGy_air_ s^−1^. The detector exhibited stable signal output following exposure to an X-ray dose of 456 Gy_air_, which is equivalent to more than 4.5 million times the dose required for a standard X-ray chest radiograph (~0.1 mGy_air_ per exposure for current commercially available instruments). This outstanding operational stability under harsh working conditions positions the detector as a compelling candidate for applications in medical diagnostics and nondestructive testing.

**Fig. 4:**
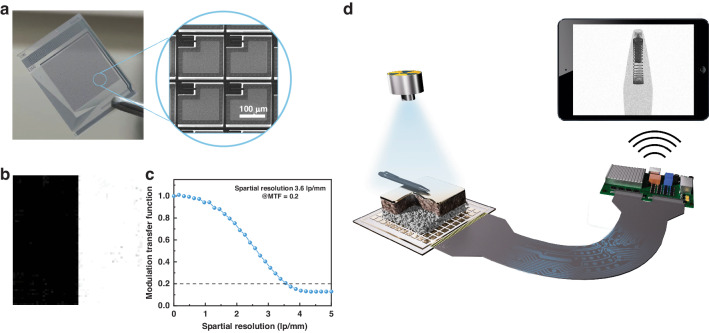
Device structure of the imaging detectors. **a** Photograph of the 64×64-pixeled TFT substrate and its microstructure; (**b**) Edge image processing for MTF calculation; (**c**) MTF curve of the TiO_2_-Q-2D FPXI; and (**d**) Schematic illustration of the X-ray imaging process. The X-ray detector structure from top to bottom is Au, Q-2D perovskite, TiO_2_ and TFT, respectively

Next, the imaging capability of the TiO_2_-Q-2D perovskite FPXI (64 × 64 pixels) was evaluated. Figure 4a presents the photograph of the 4096-pixeled TFT substrate and its microstructure. Spatial resolution is an important indicator to verify the performance and image quality of FPXI. We next measured the Modulation Transfer Function (MTF) of the imager using the slanted-edge method. A tungsten plate with a sharp edge was placed on the imager, and the edge profile was derived from the resulting X-ray image (Fig. 4b). The line spread function (LSF) was derived by differentiating the edge spread function. The MTF value was defined by the Fourier transform of LSF. Figure 4c shows the spatial resolution of our TiO_2_-2D-perovskite FPIX. To mitigate the impact of variations in pixel size and quantify the extent of signal crosstalk within the device, we employed the widely accepted metric of “line pairs per pixel” to assess the spatial resolution of the imager^37^. We surprisingly found that the TiO_2_-Q-2D perovskite FPXI exhibited an exceptional resolution of 3.6 lp mm^−1^@MTF = 0.2. The exceptional resolution of 3.6 lp mm^−1^ corresponds to 0.72 lp/pixel at a pixel size of 200 μm, which surpasses the values reported in literature for FA_0.9_MA_0.05_Cs_0.05_Pb(I_0.9_Br_0.1_)_3_ (0.46 lp/pixel), MAPbI_3_ (0.22 lp/pixel) and MAPbI_3_ (0.16 lp/pixel) FPXIs, further corroborating the reduced spatial signal crosstalk compared to 3D perovskites^10,37,38^. The higher spatial resolution of our devices can be attributed to the suppressed ion migration in perovskites and an amplified effect due to the microfocus-X-ray-source. It is well known that signal crosstalk primarily originates from charge-sharing effects between adjacent pixels^39^. In imaging devices, we share a common top electrode, so the electric field is not strictly vertical. Hence, lateral migration of ions and electrons is inevitable. Just like electronic signals, ions also experience crosstalk between neighboring pixels. For samples without ion migration, this crosstalk contribution can be eliminated, thus benefiting the attainment of better spatial resolution. Moreover, an amplified effect is always existing in the imaging system, especially when we use a microfocus-X-ray-source^40,41^. Although we conduct tests with the object placed directly on the detector, considering the use of a microfocus X-ray source and the certain thickness of the perovskite functional layer, there will still be a certain amplification effect on the signal collected by the TFT pixels.

Commercial X-ray instruments typically require high-dose imaging, so further lowering the radiation dose will reduce the health risk to patients. We evaluated the low-dose imaging properties of the TiO_2_-Q-2Q perovskite FPXI, whose structure and imaging process are shown in the Fig. 4d. The signal was transmitted via Bluetooth, which is very useful for portable use scenarios.

**Fig. 5:**
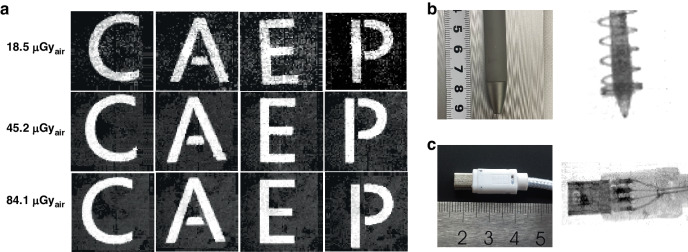
X-ray imaging properities. X-ray images of (**a**) metal letters at different dose rate, (**b**) ball-point pen, and (**c**) data line

Figure 5a present the imaging of the stainless-steel mask letters “C”, “A”, “E”, and “P” as the target object under different X-ray doses (Fig. S15). With an X-ray dose of 18.5 μGy_air_, the contour of the metal letters can already be discerned. As the X-ray dose is further increased, the clarity of the images significantly improves, and high-quality X-imaging can be obtained under an X-ray dose of ~50 μGy_air_. Typically, an X-ray dose of 100 μGy_air_ is needed to acquire a high-quality image with commercially available X-ray instruments. Thus, the X-ray dose used here are lower than that used in commercialized equipment.

We also used the TiO_2_-Q-2D perovskite FPXIs to acquire X-ray images of a ball-point and data cable adapter. As shown in Fig. 5b, c, the FPXIs can clearly represent the outline of the object under X-rays in the 64×64 matrix with gray information. Both the outline of the spring in the ball-point pen and the internal circuit of the data line are clearly visible under a low X-ray dose of 45.2 μGy_air_. These images highlight the potential value of the technology in a variety of applications. It is known that the spatial resolution of the device is largely limited by the pixel size and pixel number of the detectors, and better resolution in the X-ray imaging can be realized by increasing the pixel number and reducing the pixel size in a future study.

## Discussion

In summary, we successfully regulated the crystallization process of perovskite through the mixed atmosphere of CH_3_NH_2_ and NH_3_, and obtained high-quality Q-2D perovskite films grown from bottom to top. We further integrated Q-2D perovskite films with TiO_2_ layers to enhance the electron transport performance of the detector, ultimately achieving an ultrahigh sensitivity of 29721.4 μC Gy_air_^−1^ cm^−2^ for low-dimensional perovskite polycrystalline X-ray detector, and a low detection limit of 20.9 nGy_air_ s^−1^. The TiO_2_-Q-2D perovskite FPXI achieves a high spatial resolution of 3.6 lp mm^−1^ and produces clear X-ray images at lower doses compared to commercial devices. Further optimizing the pixel size and increasing the pixel number would facilitate practical applications in medical diagnostics and nondestructive inspection. By adjusting the film-forming mechanism of perovskite and the device structure design, we obtained a sensitive portable FPIX, which enhanced the competitiveness of low-dimensional perovskite detector as the next-generation X-ray detector.

## Materials and methods

### Materials

Ethanol (99.5%), anhydrous (99.8%), Terpineol (95%) and Ammonium chloride (NH_4_Cl, 99.99%) were purchased from Aladdin Reagent Ltd. Methylammonium iodide (MAI, 99.5%), Methylammonium Chloride (MACl, 99.5%), 1-butanaminium iodide (BAI, 99.5%) and Lead iodide (PbI_2_, 99.9%) were purchased from Xi’an p-OLED Corp. Potassium hydroxide (KOH,95%) was purchased from Macklin. Titanium dioxide nano powder (TiO_2_, P25) was purchased from Degussa. Titanium (IV) isopropoxide (98%) was acquired from J&K Scientific. TFT substrates was purchased for LinkZill Corp., and data was collected by a homemade readout circuit. All chemicals used in this work were used as received.

### Preparation of low-temperature titanium dioxide interfacial layer

Firstly, TiO_2_ precursor slurry was prepared: 0.2 g TiO_2_ nanoparticle powder was mixed with 4.5 mL terpineol and ground for 10 min, then transferred to glass bottle, 33.4 μL Titanium (IV) isopropoxide was added, ultrasonic for 30 min for reserve. The interfacial layer of TiO_2_ was prepared by scraping coating method. The ITO or TFT substrates was first treated with UV for 30 minutes, and the precursor paste was evenly applied with a scraper at the speed of 2 mm s^−1^. Finally, the wet film was placed on the 80 °C hot plate, slowly heated to 200 °C and held for 30 min to obtain the solid film.

### Preparation of perovskite layer by atmosphere-assisted method

The preparation process is shown in the schematic diagram. The preparation of liquid perovskite was referred to our previous preparation process. 400 μL acetonitrile was added to dilute liquid perovskite to obtain precursor solution. The ITO and TFT substrates with/without TiO_2_ were treated with UV for 15 min, and the 100 μL perovskite precursor was evenly applied with a scraper under a speed of 5 mm s^−1^. Then, the wet film was subsequently placed in a partially enclosed glass container containing NH_3_ and CH_3_NH_2_ gases, subjected to gradual heating using a constant temperature oven, and ultimately maintained at 100 °C for a duration of 30 min. The NH_3_ and CH_3_NH_2_ are synthesized through the combination of NH_4_Cl/MACl with KOH, resulting in the following reaction formula:

$${\rm{MACl}}/{{\rm{NH}}}_{4}{\rm{Cl}}+{\rm{KOH}}\to {{\rm{CH}}}_{3}{{\rm{NH}}}_{2}/{{\rm{NH}}}_{3}\uparrow +{{\rm{H}}}_{2}{\rm{O}}+{\rm{KCl}}$$

### Device preparation

Detectors was prepared by evaporating 80 nm-thick gold on the perovskite layer as top electrode.

### Characterization of materials

X-ray diffraction (XRD) measurements of BA_2_MA_9_Pb_10_I_31_ films were performed under Empyrean X-ray diffractometer (Cu tube with wavelengths of 1.54184 Å) with a scan rate of 2°/min. UV−vis absorption spectra were acquired in reflection mode on a Shimadzu Corporation UV-3600 spectrophotometer, utilizing BA_2_MA_9_Pb_10_I_31_ films deposited on glass substrates. Scanning electron microscope (SEM) images were captured at accelerating voltages of 5 kV (SIGMA HD ZEISS Company) and 10 kV (thermo scientific Apreo 2).

### Ion conduction measurement

The activation energy of ion conduction (*E*_a_) was measured by temperature-dependent conductivity in dark. The ionic conduction is a thermally activated process. Thus, we can find two typical regions in the temperature-dependent conductivity curves, where electronic conduction dominates the low temperature region and ion migration dominates the high temperature region. The *E*_a_ was obtained by fitting the slope of the straight line in the high temperature region using the Nernst-Einstein relationship:

$${\mathrm{ln}}\left({\sigma }_{T}T\right)={\mathrm{ln}}{\sigma }_{0}-\frac{{E}_{a}}{{k}_{B}T}$$

where, *T* is the temperature, *σ*_T_ is the conductivity, *σ*_0_ is the theory of electrical conductivity, *k*_B_ is the Boltzmann constant.

### Carrier mobilities analysis

The time of flight method (TOF) measurements were conducted by irradiating the devices with 6 ns width, 532 nm laser pulses on the Au electrode. To apply bias and detect the current of the device, we utilized the Keithley 2400 source meter and SR570 low-noise current preamplifier (Stanford Research Systems), respectively. For mobility measurements, when the laser was irradiated on the transparent ITO electrode, the transit times of electrons and holes were obtained by changing the polarity of the voltage applied to the transparent ITO electrode, and then the corresponding mobility was obtained.

### Kelvin probe force microscopy measurements

KPFM measurements were performed using amplitude modulation mode and MESP probe (Co/Cr coated) (Brucker Icon). BA_2_MA_9_Pb_10_I_31_ with several microns thickness was deposited onto ITO glass or TiO_2_/ITO substrate to prepare ITO/BA_2_MA_9_Pb_10_I_31_ and ITO/TiO_2_/BA_2_MA_9_Pb_10_I_31_ samples, respectively. The contact potential difference of the perovskite surface was measured in both dark conditions and under white light irradiation at an intensity of 20 mW cm^−2^.

### Detector performance measurement

For X-ray detection, a tungsten anode X-ray tube (Oxford Insturments’ Series 5000) was used as the source, and the Be window thickness was 127 μm. The X-ray tube was fixed with a constant of 50 kV acceleration voltage, and the operational current was tuned from 0.02 to 0.80 mA to adjust the emitted X-ray dose rate from 70 to 2726 nGy_air_ s^−1^. Dose rate calibrations of the X-ray were carried out by the 10×6-180 measurement chamber and Accu-Gold+ system (Radcal). BA_2_MA_9_Pb_10_I_31_ films detectors were measured at different voltages and the X-ray response was recorded by the low-noise current preamplifier (SR570, Stanford Research Systems).

### Signal-to-noise ratio

The noise current density (*J*_n_) is obtained from the variance of the photocurrent density ($\bar{{J}_{p}}$):

$${J}_{n}={\left[\frac{1}{N}\mathop{\sum }\limits_{i=1}^{N}{({J}_{i}-\bar{{J}_{p}})}^{2}\right]}^{1/2}$$

where *N* is the number of current sampling points.

The *J*_s_ is obtained by:

$${J}_{s}={J}_{p}-{J}_{d}$$

where *J*_d_ is the dark current density of the detector. Then, signal-to-noise ratio (SNR) is obtained by calculating the ratio of *J*_s_ to *J*_n_.

### Mean energy calculation of the continuum bremsstrahlung X-ray spectrum

The mean energy of the continuum bremsstrahlung X-ray spectrum was derived by following equation:

$$\bar{{\rm{E}}}={\int }_{0}^{{E}_{max }}p(E){dE}$$

*p*(*E*) is the distribution probability of X-rays with the energy of *E*.

$$\bar{{\rm{E}}}=\sum _{i}p({E}_{i})\triangle {E}_{i}$$

where $p({E}_{i})$ and ∆*E*_*i*_ are the distribution probability and the energy bin width of the X-rays at the energy bin. For the tube operating at 50 kV tube voltage, the mean energy is calculated to be 42 keV with a 2.6 cm quartz glass filter layer).

### TRPL measurement

The PL decays were fitted with a biexponential function containing both a fast and slow decay process using Equation:

$$f\left(t\right)={A}_{1}{e}^{\frac{-t}{{\tau }_{1}}}++{A}_{2}{e}^{\frac{-t}{{\tau }_{2}}}+{A}_{3}{e}^{\frac{-t}{{\tau }_{3}}}+C$$

Where, A_n_ and *τ*_n_ represent the amplitude and delay time/lifetime of the n th component (here, *n* = 1,2,3), respectively, and C is a constant that represents the baseline offset. The average life can then be calculated by the following formula:

$${\tau }_{{ave}}=\frac{{A}_{1}{{\tau }_{1}}^{2}+{A}_{2}{{\tau }_{2}}^{2}+{A}_{3}{{\tau }_{3}}^{2}}{{A}_{1}{\tau }_{1}+{A}_{2}{\tau }_{2}+{A}_{3}{\tau }_{3}}$$

### Calculation of modulation transfer function

The modulation transfer function (MTF) was calculated by the slanted-edge method. Sharp-edge X-ray imaging was obtained on the standard tungsten plate. Then the edge spread function (ESF(x)) was derived from the edge image. The line spread function LSF(x) was the derivation of the ESF(x) and the MTF(ν) was the Fourier transform of LSF(x) by the following equation:

$$MTF(\nu)=F(LSF(x))=F\left(\frac{dESF(x)}{dx}\right)$$

where *ν* represents the spatial resolution and *x* means the position of the pixels. The spatial resolution of the X-ray imager was derived when the MTF value decreases to 0.2.

### Supporting information

Figures S1–S15 show the XRD patterns, absorption features, activation energy for ionic migration, μτ product test, TOF transient curves, X-ray absorption coefficients, TRPL results, dose rate-dependent signal current, bias-dependent sensitivity, X-ray response under 0 bias, device operational stability, and photograph of the objects for X-ray imaging tests, and Table S1 shows performance comparison of polycrystalline X-ray detectors reported in literature.

### Supplementary information

Supplementary Information for Bottom-up construction of low-dimensional perovskite thick films for high-performance X-ray detection and imaging
