# Supplementary material for: Bottom-up construction of low-dimensional perovskite thick films for high-performance X-ray detection and imaging
Source: Light Sci Appl. 2024 Jul 23;13:174. doi: 10.1038/s41377-024-01521-2 (PMC11266548; doi:10.1038/s41377-024-01521-2)
Supplement: Supplementary file 1 — Supplementary Information for Bottom-up construction of low-dimensional perovskite thick films for high-performance X-ray detection and imaging [file 41377_2024_1521_MOESM1_ESM.docx]

**Supplementary Information for**

**Bottom-up construction of low-dimensional perovskite thick films for high-performance X-ray detection and imaging**

Siyin Dong^1^, Zhenghui Fan^1^, Wei Wei^2^, Shujie Tie^1^, Ruihan Yuan^1^, Bin Zhou^1^, Ning Yang^1^, Xiaojia Zheng^1,^* , Liang Shen^2,^*

^1^ Sichuan Research Center of New Materials, Institute of Chemical Materials, China Academy of Engineering Physics, Shuangliu, Chengdu 610200, China

^2^ State Key Laboratory of Integrated Optoelectronics, College of Electronic Science and Engineering, International Center of Future Science, Jilin University, Changchun 130012, China

Email: shenliang@jlu.edu.cn; xiaojia@caep.cn

KEYWORDS: crystallization regulation, perovskite, X-ray imaging, spatial resolution, sensitivity

**
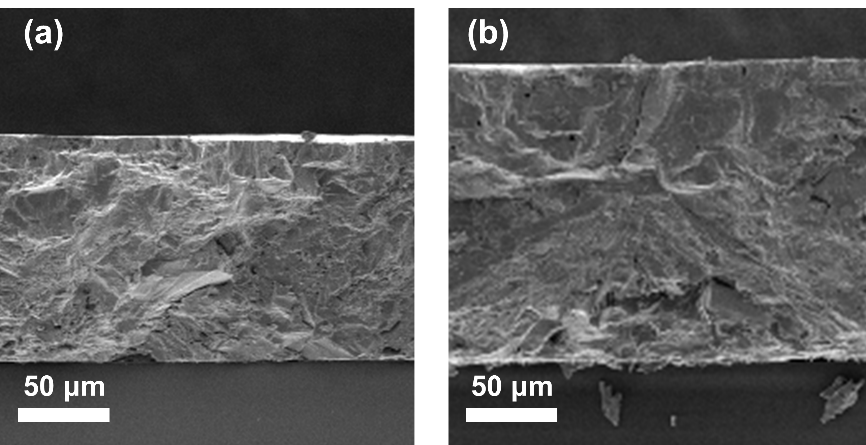
**

**Figure S1.** The cross-section SEM images for BA_2_MA_9_Pb_10_I_31_ films with different thickness prepared by mixed atmosphere of CH_3_NH_2_ and NH_3_.


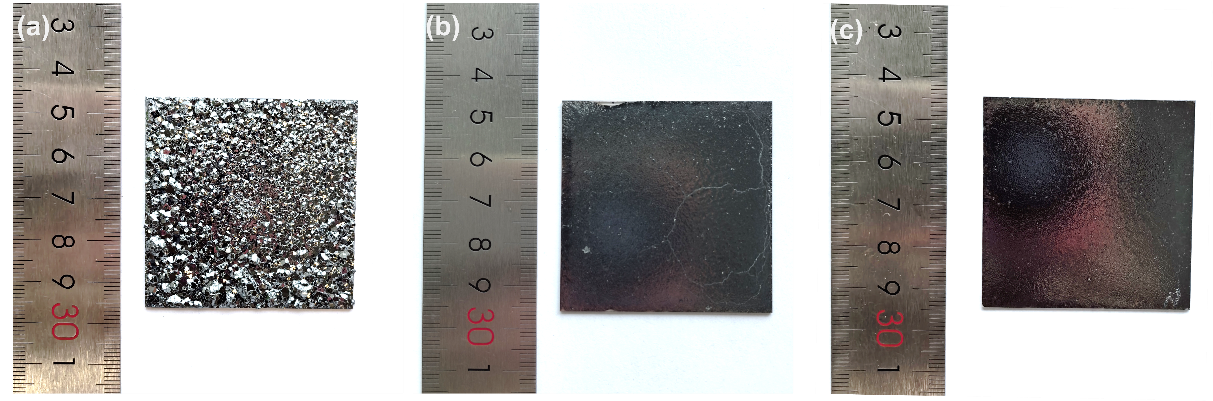


**Figure S2.** Photograph of large area perovskite film prepared under (a) Control, (b) CH_3_NH_2_ and (c) CH_3_NH_2_/NH_3_ atmosphere. The suboptimal quality of Control can be ascribed to the premature crystallization of perovskite on the precursor solution's surface, which hindered solvent evaporation. This resulted in a rapid solvent volatilization during the annealing phase, thereby undermining the film's surface integrity. However, when CH_3_NH_2_ is utilized to suppress surface crystallization, the perovskite crystals exhibit a bottom-up growth pattern, significantly improving the film's quality as illustrated in Figure b. Furthermore, the crystallization kinetics of the perovskite are modulated by NH_3_, yielding a more uniform film surface and enhancing the specular properties. (The mobile phone can be reflected in the membrane surface in Figure c).


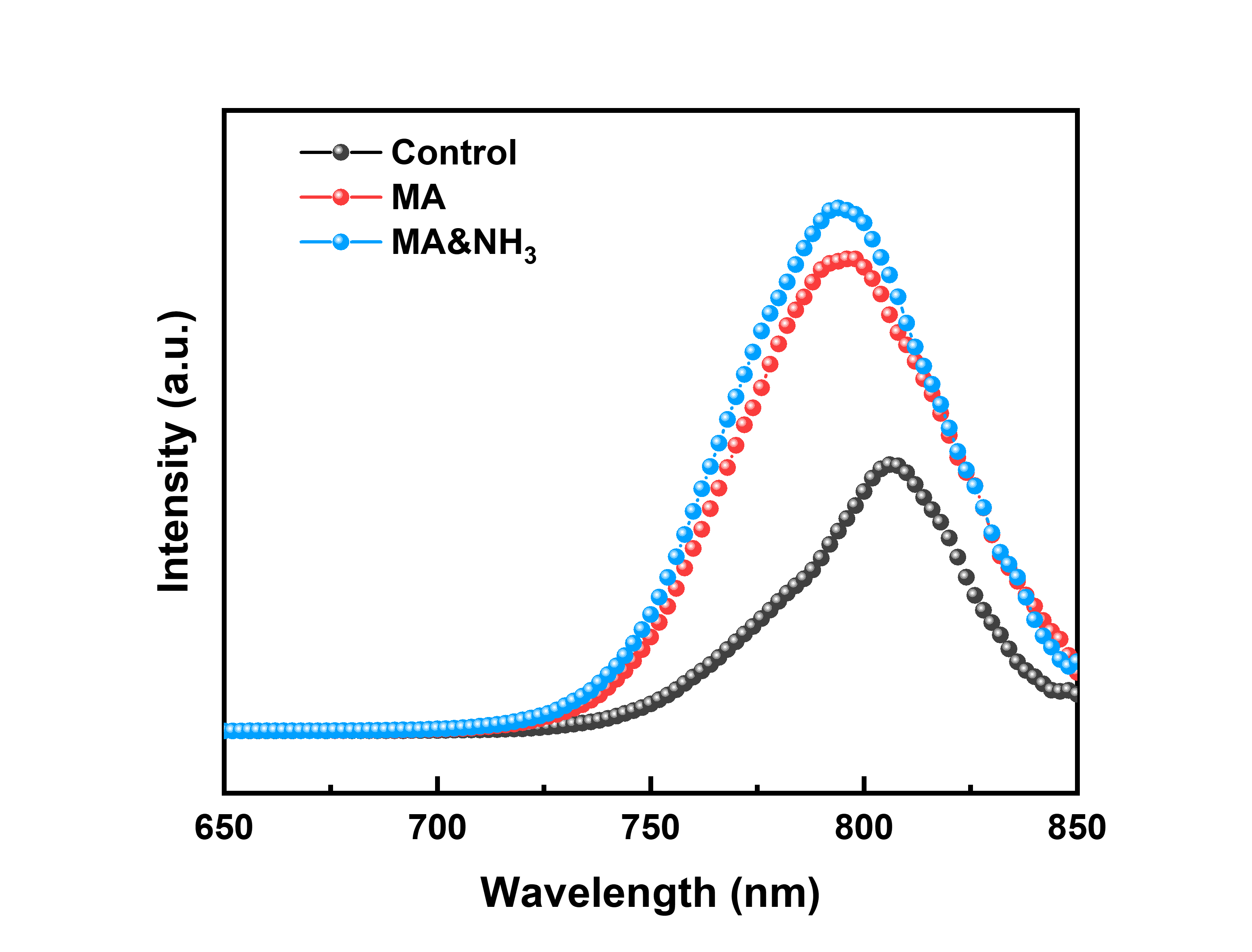


**Figure S3.** Steady PL curves of perovskite films prepared under (a) Control, (b) CH_3_NH_2_ and (c) CH_3_NH_2_/NH_3_ atmosphere. The perovskite film prepared by mixed atmosphere shows the strongest PL intensity (with peak at 794 nm) with a notably blue-shifted peak compared to the control sample (with peak as 805 nm), indicating that atmosphere-controlled crystallization can improve the quality of the films and reduce the non-radiative recombination.

**Figure S4.** XRD of NH_4_PbI_3_ perovskite film prepared by DMF, BA_2_MA_9_Pb_10_I_31_ film before and after NH_3_ treatment, and BA_2_MA_9_Pb_10_I_31_ film prepared in NH_3_-rich atmosphere. Notably, the perovskite synthesized in an NH_3_-rich atmosphere exhibited diffraction peak corresponding to NH_4_PbI_3_, indicating that NH_3_ can incorporate into the lattice and form the NH_4_PbI_3_ phase. However, no diffraction peak corresponding to the NH_4_PbI_3_ was observed in the perovskite film prepared under a mixed atmosphere, indicating the complete remove of NH_3_ following annealing. Therefore, mixing a certain proportion of NH_3_ can effectively delay the crystal growth rate to improve the quality of perovskite film by forming the NH_4_PbI_3_ intermediate phase.


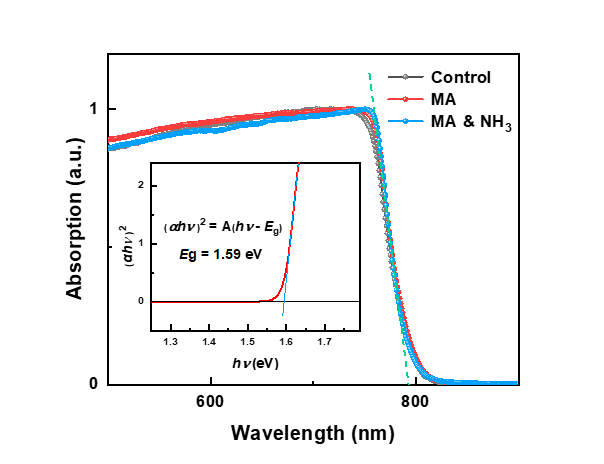


**Figure S5.** Absorption spectrum of BA_2_MA_9_Pb_10_I_31_ films. All BA_2_MA_9_Pb_10_I_31_ films have similar band gaps, which is same as the XRD results, indicating that no NH_4_PbI_3_ remains after annealing.


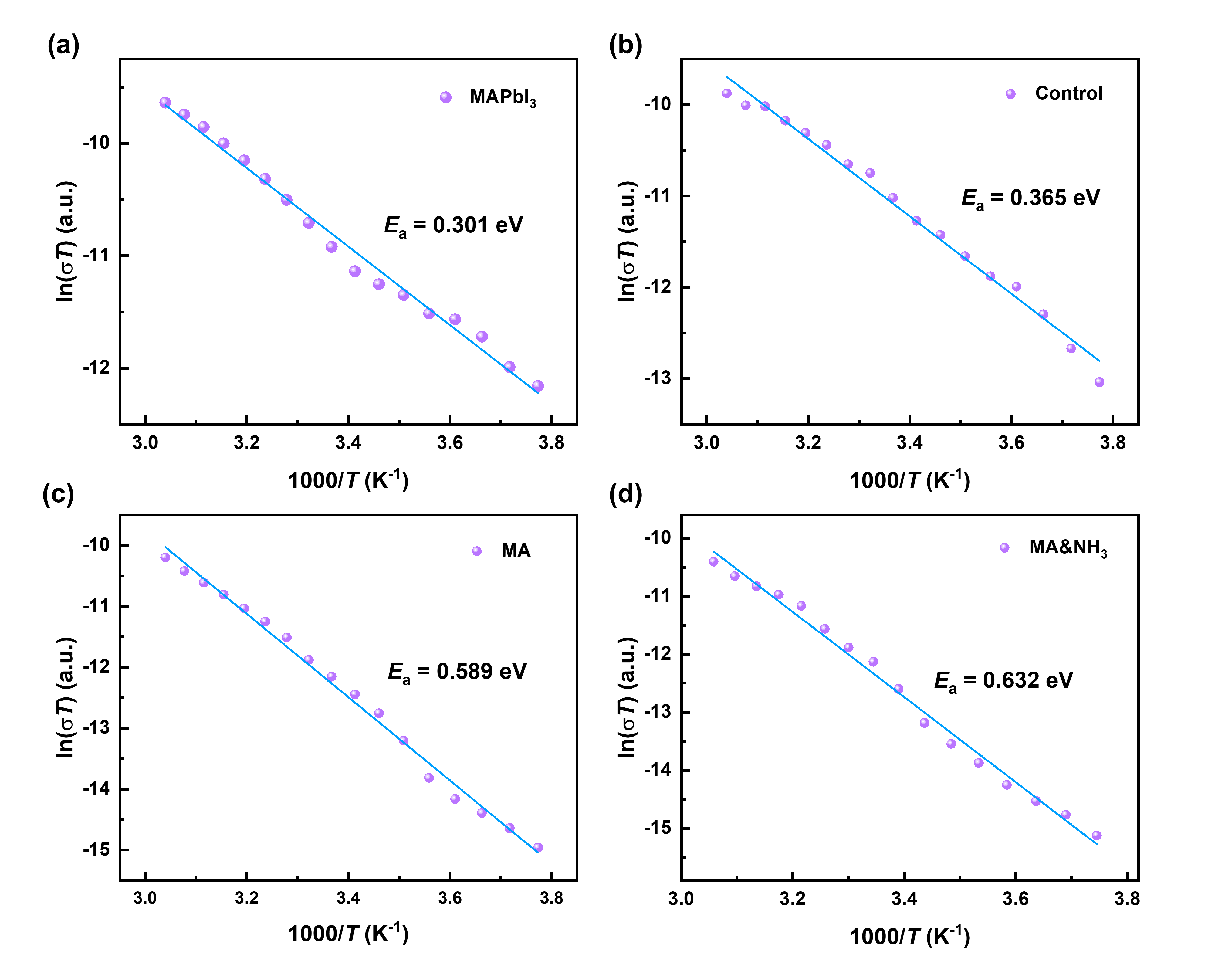


**Figure S6.** Temperature-dependent conductivity of (a) MAPbI_3_, and (b) Control, (c) CH_3_NH_2_, (4) CH_3_NH_2_/NH_3_ of BA_2_MA_9_Pb_10_I_31_ perovskite detectors.


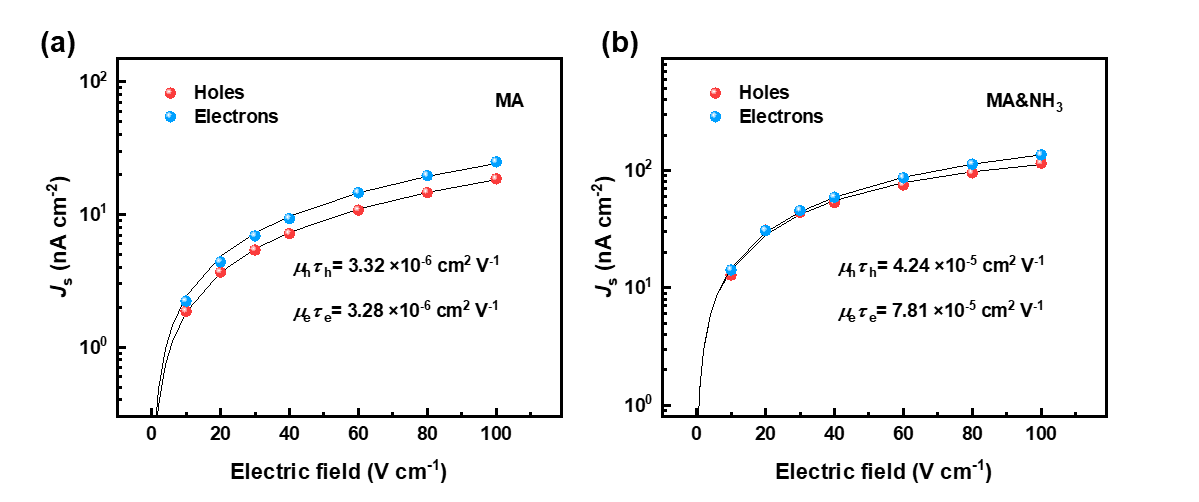


**Figure S7.** Bias-dependent photo-induced signal current and corresponding carrier mobility-lifetime (*μτ*) product for BA_2_MA_9_Pb_10_I_31_ films with (a) CH_3_NH_2_ and (b) CH_3_NH_2_ /NH_3_ atmosphere. The quality of the film under Control and NH_3_ atmosphere is poor, and *μτ* cannot be obtained.


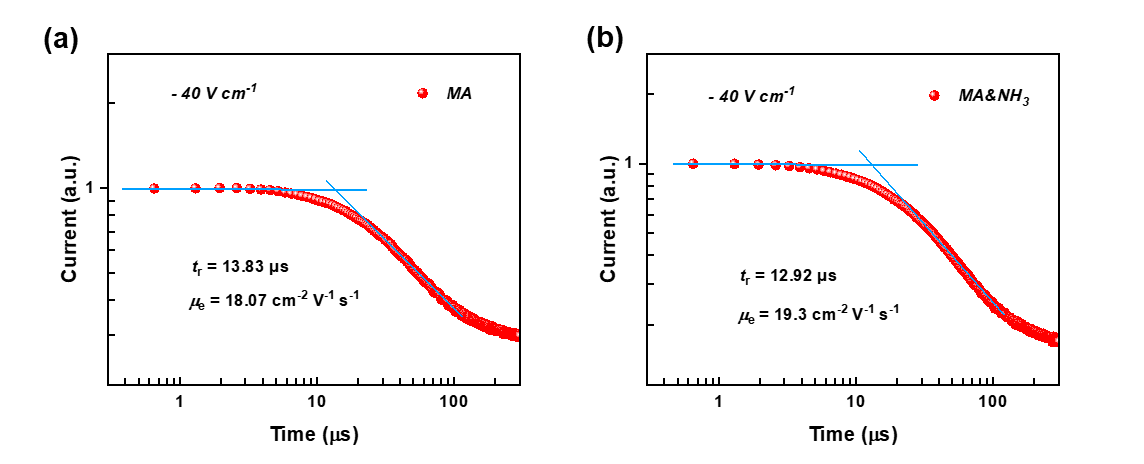


**Figure S8.** TOF transients of the photo generated carriers in (a) CH_3_NH_2_ (b) CH_3_NH_2_/NH_3_ atmosphere prepared perovskite under -40 V cm^-1^.


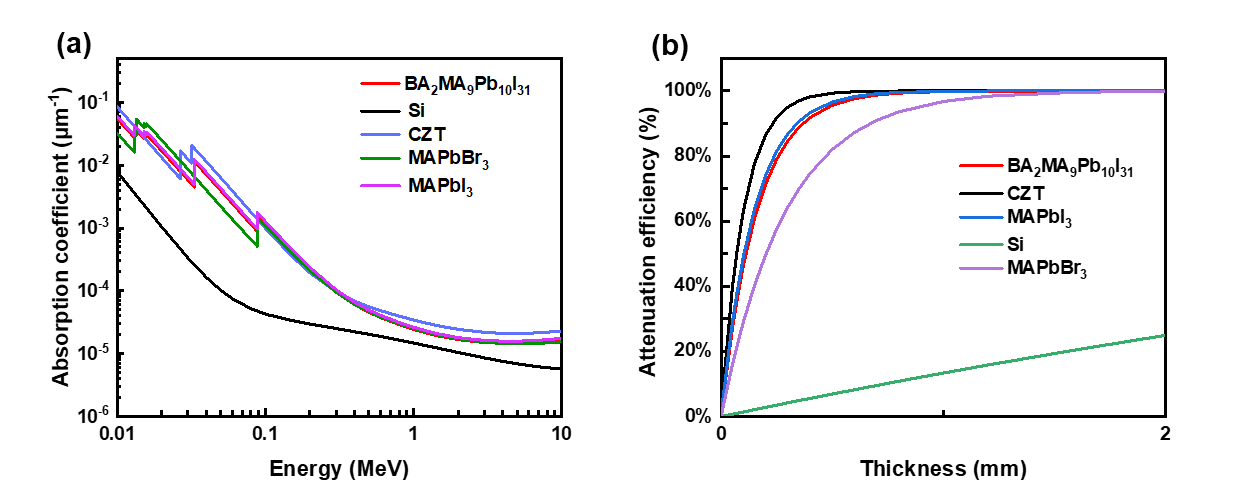


**Figure S9.** (a) Absorption coefficients of BA_2_MA_9_Pb_10_I_31_, Si, CZT, MAPbBr_3_ and MAPbI_3_ as a function of photon energy. The absorption spectra were obtained from the photon cross-section database.^1^ (b) Thickness-dependent X-ray attenuation efficiency for 42 keV X-rays (mean energy of the X-ray photons used in this work) of BA_2_MA_9_Pb_10_I_31_, Si, CZT, MAPbBr_3_ and MAPbI_3_.

_
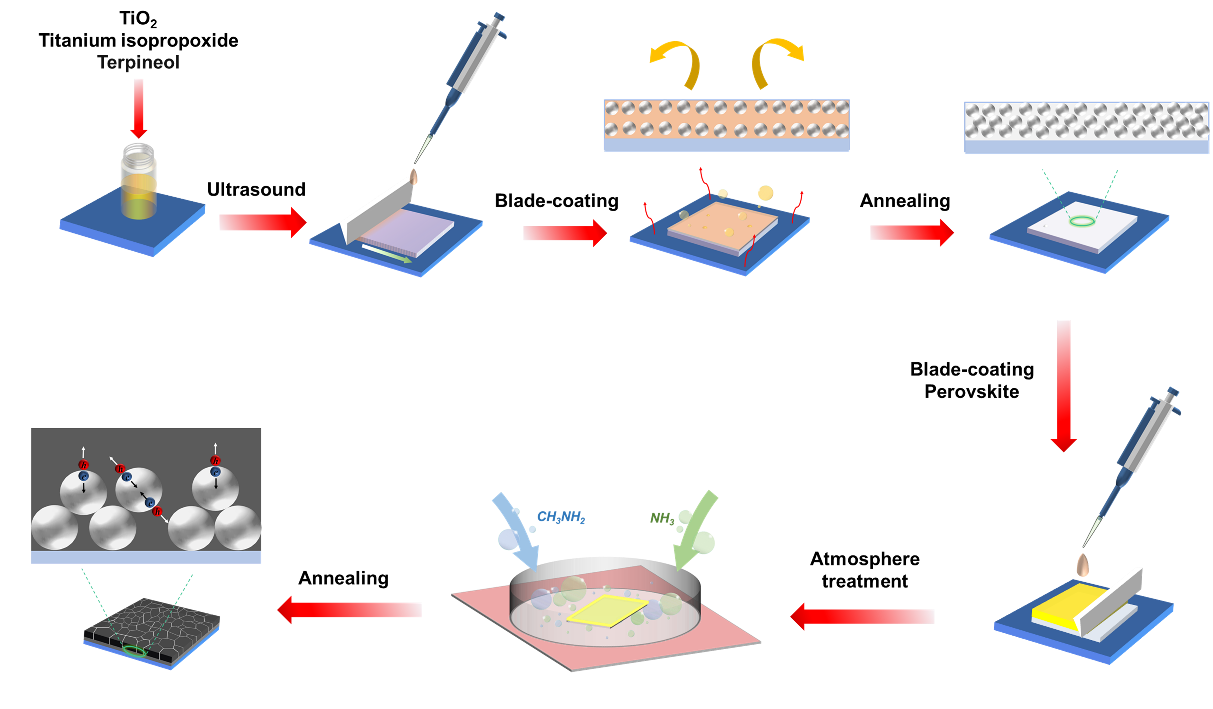
_

**Figure S10.** Schematic diagram of TiO_2_/ perovskite heterojunction preparation.


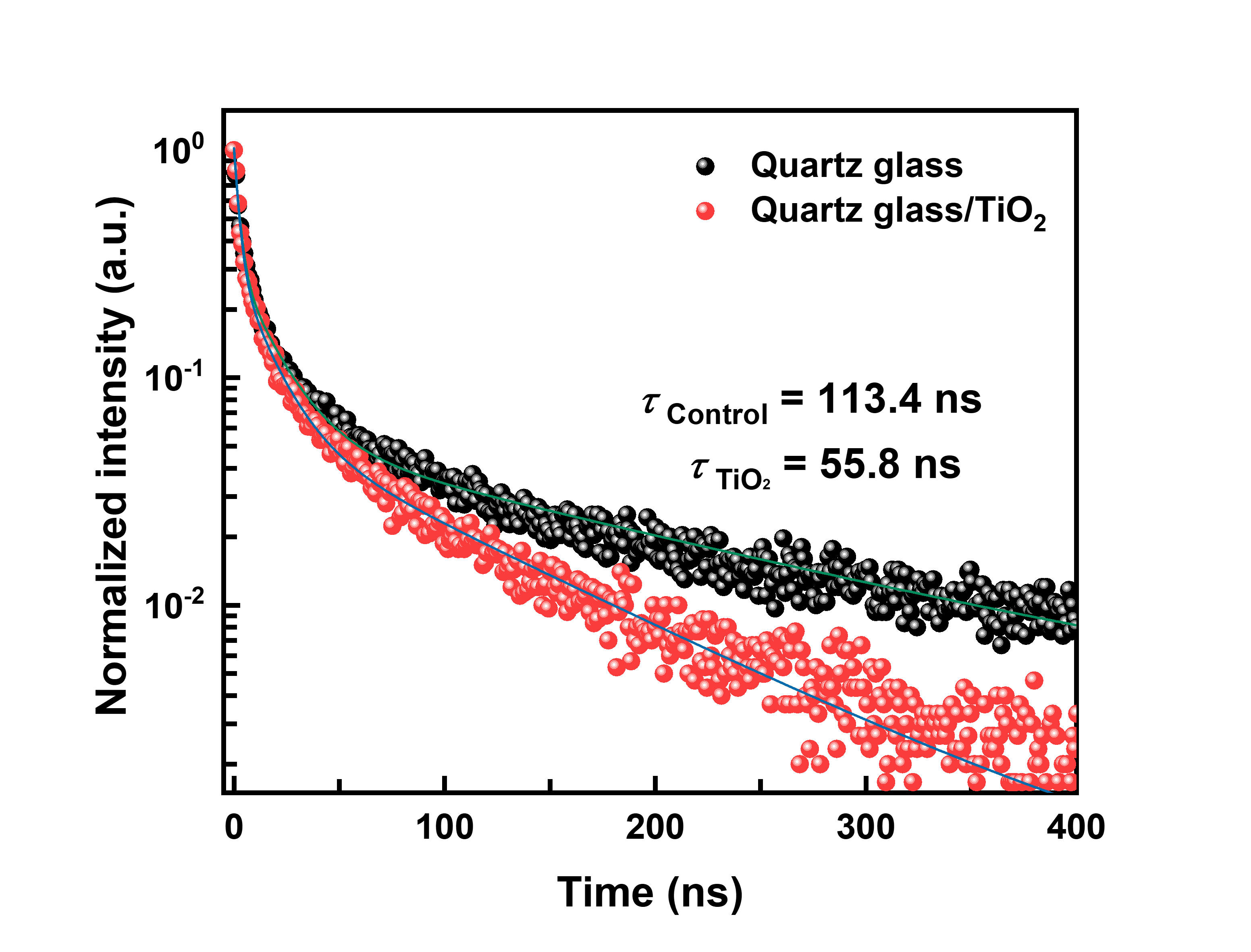


**Figure S11.** TRPL of BA_2_MA_9_Pb_10_I_31_ perovskite films on different substrates. The samples for TRPL characterization were prepared by depositing BA_2_MA_9_Pb_10_I_31_ onto quartz glass and TiO_2_/quartz glass substrates, respectively.


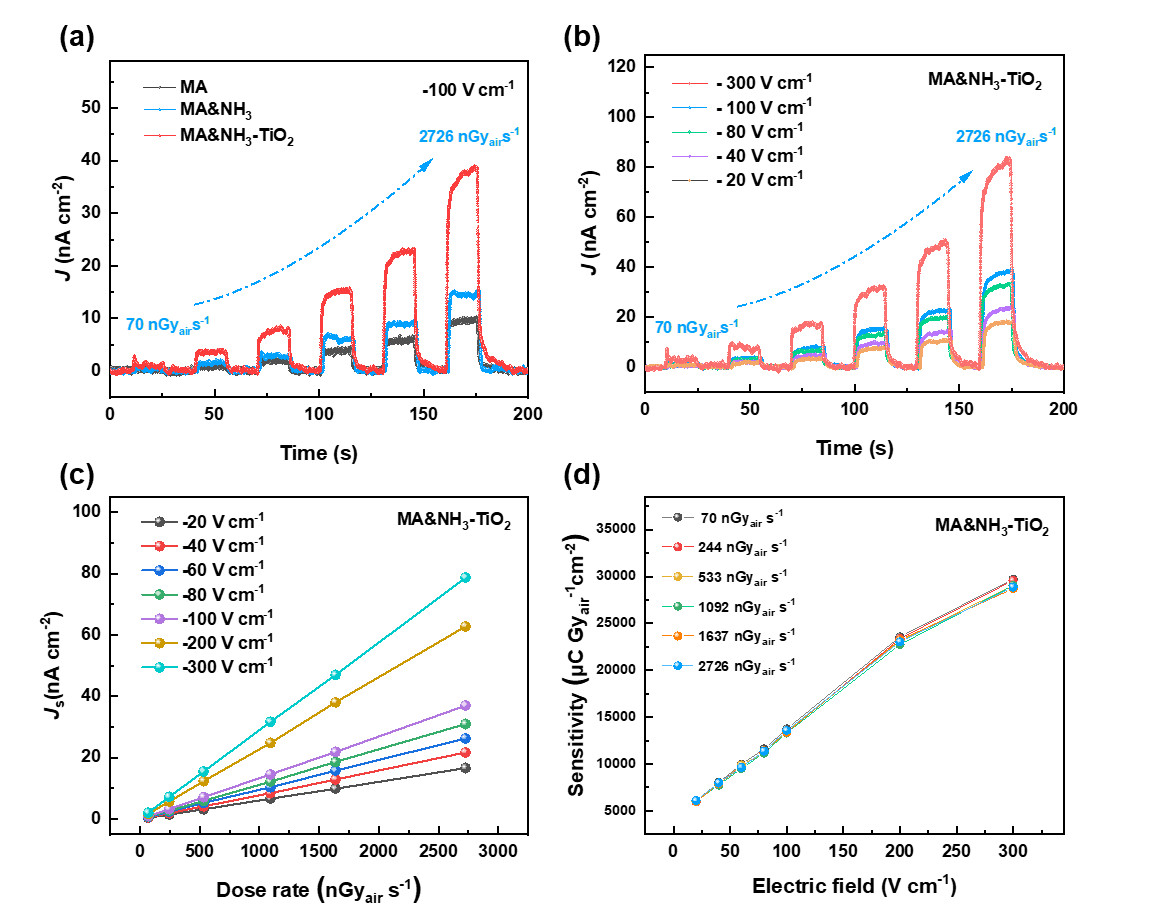


**Figure S12.** (a) X-ray response of various detectors under an applied electric field of -100 V cm^-1^, (b) X-ray response of TiO_2_-Q-2D perovskite X-ray detector under varying electric fields, (c) Dose rate-dependent signal current of TiO_2_-Q-2D perovskite X-ray detector, and (d) Bias-dependent sensitivity under varying X-ray dose rates.


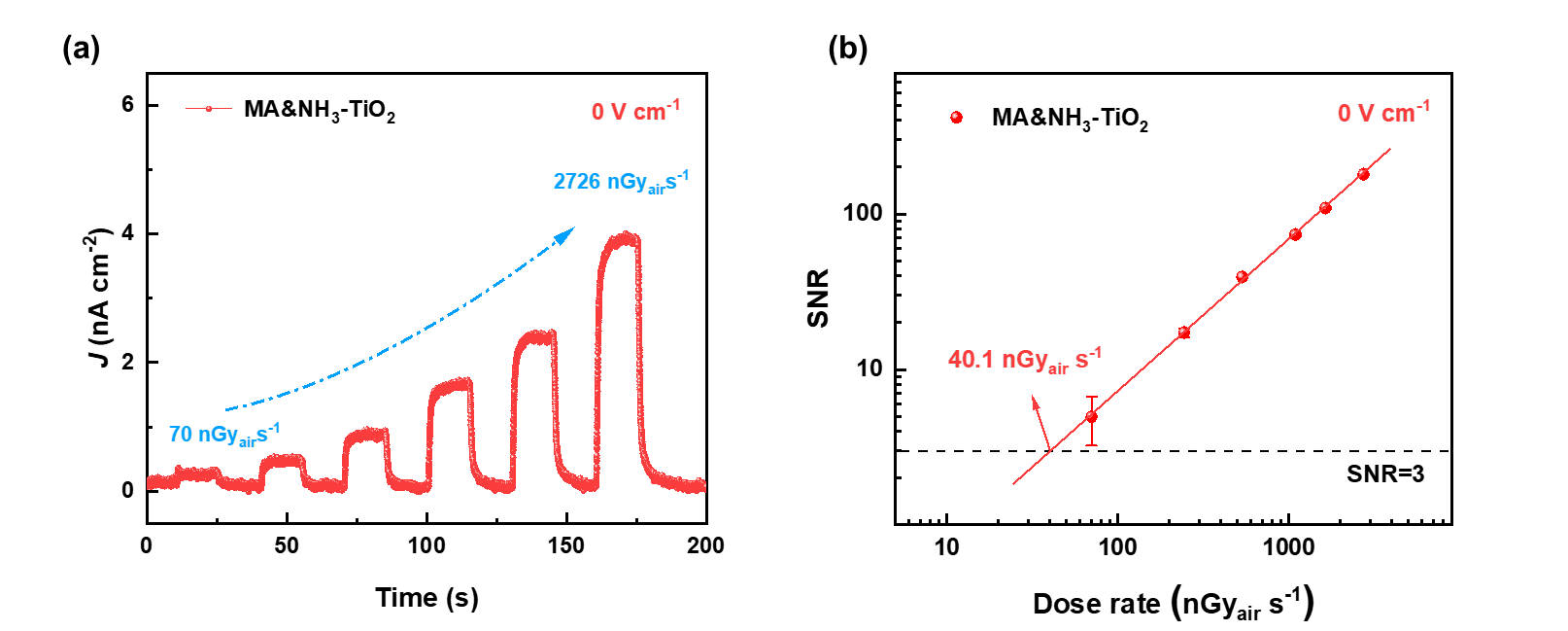


**Figure S13.** (a) X-ray response and (b) X-ray dose rate dependent SNR of the TiO_2_-Q-2D perovskite X-ray detector without external electric field (0 V cm^-1^)


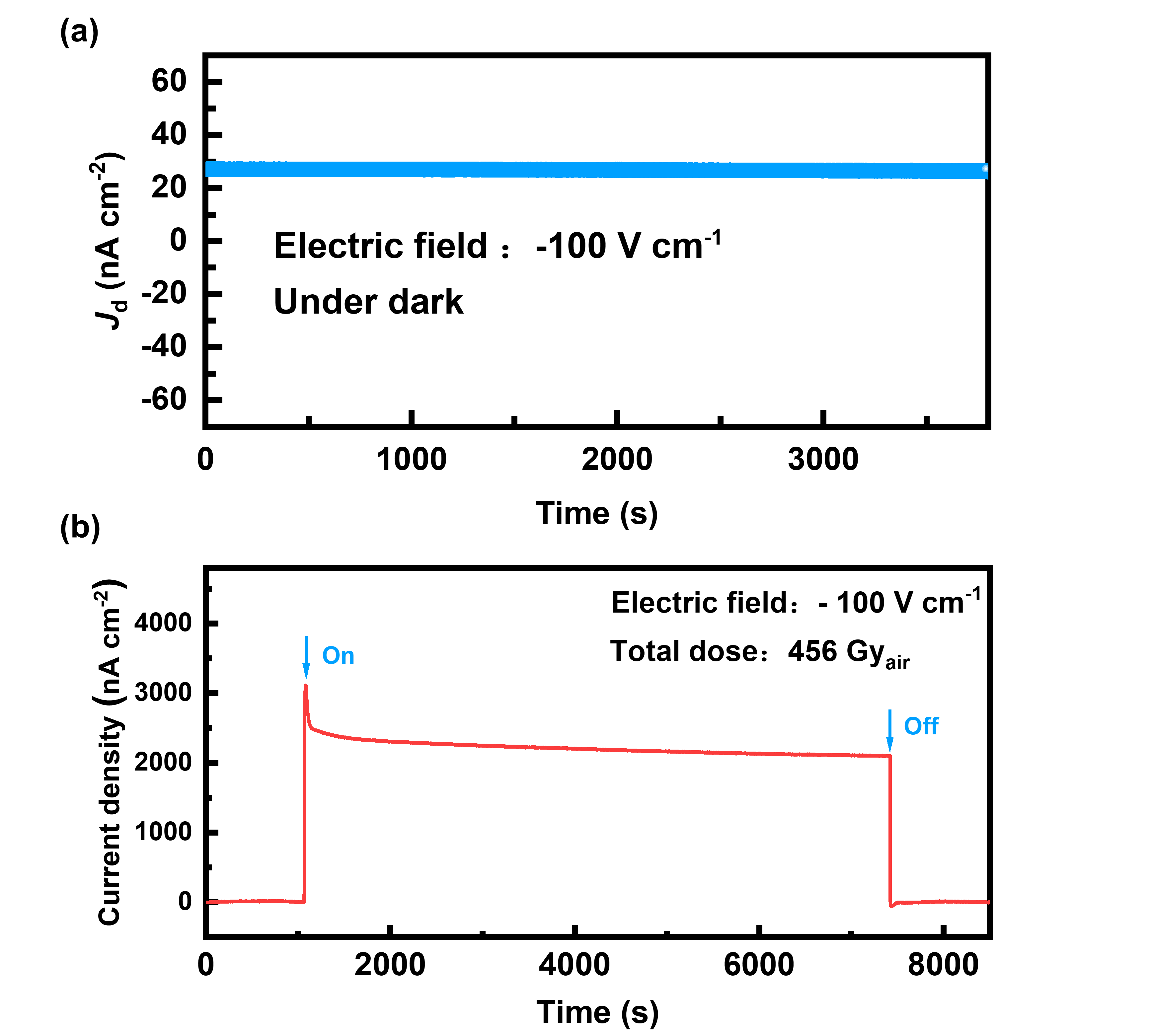


**Figure S14.** (a) Device operational stability of the TiO_2_-2D perovskite detectors at -100 V cm^-1^, (b) Device operational stability under a bias of -100 V cm^-1^ for the TiO_2_-2D perovskite detector under continuous X-ray irradiation with a total X-ray dose of 456 Gy_air_.


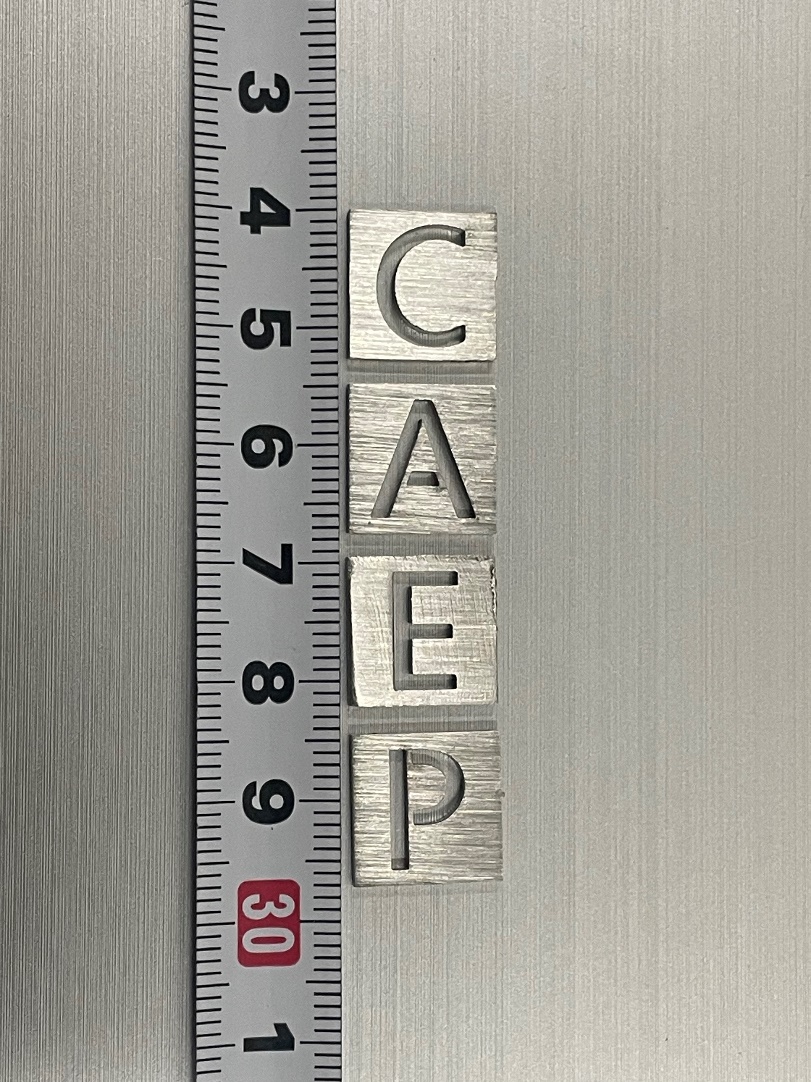


**Figure S15.** Photograph of the “CAEP” letters for X-ray imaging.

**Table** **S1.** Performance comparison of Polycrystalline X-ray detectors reported in literature.

| Materials | Dimension | Bias field  （V mm^-1^） | LoD  (nGy_air_ s^-1^) | Sensitivity  (μC Gy_air_^-1^ cm^-2^) | Material form | Ref. |
| --- | --- | --- | --- | --- | --- | --- |
| a-Se | - | 10000 | 5500 | 20 | Polycrystalline | 2 |
| MA_3_Bi_2_I_9_ | 0D | - | 2.71 | 2065 | Polycrystalline | 3 |
| Cs_3_Bi_2_Br_3_I_6_ | 2D | 200 | 10700 | <1 | Polycrystalline | 4 |
| Cs_3_Bi_2_I_9_ | 0D | 120 | 231 | 368 | Polycrystalline | 5 |
| MAPbI_3_ | 3D | 160 | 28.57 | 22400 | Polycrystalline | 6 |
| MAPbI_3_ | 3D | 44 | 12.4 | 12382 | Polycrystalline | 7 |
| (F-PEA)_3_BiI_6_ | 2D | 100 | 1800(//); 30(⊥) | 118.6(//); 52.6(⊥) | Polycrystalline | 8 |
| PEA_2_PbBr_4_ | 2D | 4000 | 42 | 806 | Polycrystalline | 9 |
| MAPbI_3_/  (PEA)_2_MA_3_Pb_4_I_13_ | 2D/3D | 33.3 | 480 | 19500 | Polycrystalline | 10 |
| (BA_2_PbBr_4_)_0.5_FAPbI_3_ | 2D/3D | - | 4.2 | 13600 | Polycrystalline | 11 |
| PEA_2_MA_8_Pb_9_I_28_ | 2D | 600 | 10 860 | 69 | Polycrystalline | 12 |
| BA_2_MA_9_Pb_10_I_31_ | 2D | 110 | 8.1 | 5362 | Polycrystalline | 13 |
| PentA_2_MA_9_Pb_10_I_31_ | 2D | 210 | 7.8 | 6800 | Polycrystalline | 14 |
| **BA_2_MA_9_Pb_10_I_31_** | 2D | 30 | 20.9 | 29721 | Polycrystalline | **This**  **work** |

**Supplementary References:**

1. M.J. Berger. et al. *XCOM: Photon cross sections database: NIST standard reference database 8 (XGAM)* [*https://www.nist.gov/pml/xcom-photoncross-sections-database*](https://www.nist.gov/pml/xcom-photoncross-sections-database) (2013).

2. Wei, H.T. & Huang, J. S. Halide lead perovskites for ionizing radiation detection. *Nature Communications* **10**, 1066 (2019).

3. Liu, X. M. et al. Molecular doping of flexible lead-free perovskite-polymer thick membranes for high-performance X-ray detection. *Angewandte Chemie International Edition* **61**, e202209320 (2022).

4. Daum, M. et al. Self-healing Cs_3_Bi_2_Br_3_I_6_ perovskite wafers for X-ray detection. *Advanced Functional Materials* **31**, 2102713 (2021).

5. Wei, S. Y. et al. Enhanced carrier transport in X-ray detector based on Cs_3_Bi_2_I_9_/mxene composite wafers. *Advanced Optical Materials* **10**, 2201585 (2022).

6. Song, Z. H. et al. Rheological engineering of perovskite suspension toward high-resolution X-ray flat-panel detector. *Nature Communications* **14**, 6865 (2023).

7. Zhang, M. et al. Solvent free laminated fabrication of lead halide perovskites for sensitive and stable X-ray detection. *The Journal of Physical Chemistry Letters* **12**, 6961-6966 (2021).

8. Li, M. B. et al. Oriented 2D perovskite wafers for anisotropic X-ray detection through a fast tableting strategy. *Advanced Materials* **34**, 2108020 (2022).

9. Lédée, F. et al. Ultra-stable and robust response to X-rays in 2D layered perovskite micro-crystalline films directly deposited on flexible substrate. *Advanced Optical Materials* **10**, 2101145 (2022).

10. Xu, X. W. et al. Sequential growth of 2D/3D double-layer perovskite films with superior X-ray detection performance. *Advanced Science* **8**, 2102730 (2021).

11. Peng, J. L. et al. Ion-exchange-induced slow crystallization of 2D-3D perovskite thick junctions for X-ray detection and imaging. *Matter* **5**, 2251-2264 (2022).

12. He, X. et al. Quasi-2D perovskite thick film for X-ray detection with low detection limit. *Advanced Functional Materials* **32**, 2109458 (2022).

13. Zhang, M. et al. Methylamine-assisted preparation of Ruddlesden-Popper perovskites for stable detection and imaging of X-rays. *Advanced Optical Materials* **10**, 2201548 (2022).

14. Xin, D. Y. et al. A-site cation engineering of Ruddlesden–Popper perovskites for stable, sensitive, and portable direct conversion X-ray imaging detectors. *The Journal of Physical Chemistry Letters* **13**, 11928-11935 (2022).
